# Were we happy and we didn’t know it? A subjective dynamic and financial assessment pre-, during and post-COVID-19

**DOI:** 10.1007/s10198-022-01506-1

**Published:** 2022-08-19

**Authors:** Gabriela-Mihaela Mureșan, Viorela-Ligia Văidean, Codruța Mare, Monica Violeta Achim

**Affiliations:** 1 Department of Finance, Faculty of Economics and Business Administration, Babeş-Bolyai University, Teodor Mihali Street, no. 58-60, 400591 Cluj-Napoca, Romania; 2 Department of Statistics-Forecasts-Mathematics, Faculty of Economics and Business Administration, Babes-Bolyai University, Teodor Mihali Street, no. 58-60, 400591 Cluj-Napoca, Romania; 3 Interdisciplinary Centre for Data Science, Babes-Bolyai University, 68, Avram Iancu str., 4th floor, 400083 Cluj-Napoca, Romania

**Keywords:** COVID-19, Financial stability, Happiness, Well-being, I15, I31, J17

## Abstract

The COVID-19 pandemic has brought many changes into people’s lives. Fear, job insecurity, changes in their financial stability, concerns about their future lives have changed the entire lives of people and have affected the cognitive well-being of individuals. The purpose of the present analysis is to measure how the COVID-19 pandemic, along with financial factors, has affected the perceived level of well-being of individuals. We are also interested whether there are differences between *life before* COVID-19, *life now* with COVID-19*,* and *life after* the COVID-19 pandemic, in terms of future expectations. To address this objective, we performed an ANOVA approach and a GLM estimate on repeated measures for a large sample (1572 respondents) from 43 worldwide countries, during the period May 2020 and July 2021. Our results show that financial factors reflected by both the size of income and changes in personal or family income affect the levels of happiness. Robustness checks using stress as an alternative estimator for happiness have consolidated our results. Additionally, we find that well-being during COVID-19 compared to the previous period decreased, while in future, people expect to be happier, but not more than in the past when they did not know about the existence of this virus. This is one of the first studies to investigate the relationship between happiness and income before, during, and after COVID-19. These findings are important for policymakers to improve the conditions of living in the areas of health and financial stability.

**Supplementary Information:**

The online version contains supplementary material available at 10.1007/s10198-022-01506-1.

## Introduction

The COVID-19 pandemic has brought many changes in people’s lives. First, fear and concern have appeared with the outbreak of a global pandemic and they seem to influence the cognitive well-being of every individual [66, 70, 74], but post-pandemic transformation, also means embracing uncertainty [51]. Second, job insecurity due to COVID-19 has been indirectly associated with depression and anxiety symptoms [4, 29, 42, 84]. It is clear to everyone that the consequences are multiple and complex in such a pandemic and that the financial or psychological impact becomes almost impossible to measure entirely.

The main COVID-19 lockdown policies, such as remote work, telework, and school and childcare closures, have had an indirect effect on the general happiness degree of the population. In a recent paper in Romania, Stănculescu [78] showed how positive psychology approach in the study of fear of COVID-19, highlights a negative relationship among happiness and fear in the context of the COVID-19 pandemic. Our study explores how two patterns of subjective well-being (SWB), financial stability and happiness, have or have not changed during the COVID-19 lockdown. Our first goal is to investigate whether there are differences between *life before* COVID-19, *life now* with *COVID-19,* and *life after* the COVID-19 pandemic, in terms of expectations for the future.

The purpose of the present analysis is to measure how the COVID-19 pandemic, along with financial factors, has affected the perceived level of well-being of individuals. To address this objective, we perform an ANOVA approach and a GLM estimation on repeated measures for a large sample of 1572 international respondents. Our results show the significant impact of the COVID-19 pandemic, in all forms of financial estimations. Additionally, financial factors have contributed to changes in well-being.

The remainder of this article is structured as follows: **“**Literature review” section presents the literature review, “Data and methodology” section describes the methodology, sample, and data used within our research, while “Results and discussions” section reveals the results and the related discussions. The paper ends with conclusions and limitations of our research.

## Literature review

*What is happiness? Am I happy?* These are the only two questions that many of us have asked ourselves. In the literature, happiness is defined as: 'best possible life' by Kilpatrick and Cantril [44], or 'positive emotional state' by Kitayama et al. [46], or 'highly valued matter' by Veenhoven [81]. Starting from these examples of defining the concept, we notice that the concept of happiness is a subjective one, in line with Gilbert [31]. Instead, Layard [50] argues that the meaning of happiness is the same to all people. Over time, this topic has been conceptualized under different phrases, such as happiness, well-being, or life satisfaction, depending on the purpose of the research and the cultural context.

The relationship between happiness and *mental health difficulties* caused by COVID-19 received significant attention from researchers. *Mental health difficulties* are investigated in different forms, such as *anxiety symptoms* [17, 26, 52, 54, 75, 87], *depression symptoms* [8, 30, 73], *resilience* ([40], [43] *burnout* [85, 90], *suicidal behavior*) [15], [33]. On the other hand, researchers, such as Yıldırım & Güler [89], O'Connor et al., [64] have been concerned with the *psychology of happiness* in the COVID-19 context.

According to O'Connor et al. [64], mental health and well-being of adults in the United Kingdom in the first six weeks of lockdown have been affected in a profound and long-lasting way. Furthermore, the rate of suicidal ideation increased to 14% and men appear to report higher levels of well-being compared to women. Then, Datu & Fincham [17] tested adaptability to situations (TMG dimensions), meaning in life and relatedness needs on pandemic-related perceived mental health and anxiety, in the United States and the Philippines.

Long [54] addresses the level of happiness as a dependent variable. The study evaluates the pandemic with its financial effects (like changes in employment status or household income), negative non-financial effects (the individuals’ feelings of being bored or lonely, having trouble sleeping, fighting anxiety and other people) and positive non-financial effects (increased free time for oneself or families and decreased pollution), while controlling for gender, age, income, living arrangement, and regions, for six sampled countries.

Regarding well-being, Bakkeli [6] estimates subjective well-being as a function of self-reported health, also building an ill-health dummy, for more than 3000 Norwegian employees, before the pandemic (2019) and during its early stages (2020). This paper also considers the employees’ worsened work situation, income loss due to the current pandemic, both physical and mental health risks, and the work-life conflict. It controls for gender, individual income, attained education, type of household and employment sector as well. According to the results of this survey-based research, people with poorer health are more likely to experience aggravated work situations, further related to decreased life satisfaction, through the pandemic. Behar-Zusman et al. [7] also validates the different types of household structure as valuable explanatory variables of life satisfaction during the pandemic. They also estimate a higher effect of the pandemic on individuals with low socioeconomic statuses or on single parents. Another study that covers the pre-pandemic and during the pandemic timelines is that of Engels et al. [23], whose results emphasize the important role played by sports and exercises throughout the pandemic, as a protective factor against mental health disorders, while controlling for sociodemographic factors, such as age, gender, and educational degree. Zuo et al. [93] also emphasize the importance of physical activities of various categories and frequencies during pandemic home isolation for subjective well-being of the surveyed Chinese, controlling for marital and employment status, education, gender, body mass index (BMI), age (low for people below the age of 29 and high for people above 29), household income and home ownership.

Özmen et al. [65] study the relationship between the fear of COVID-19, well-being, and life satisfaction of individuals living in Turkey. Concerning their fear of COVID-19, there are differences given by educational levels, gender, working status, age, having any chronic diseases, and income levels as well. Results of the regression analysis emphasize the fact that: 'the fear of COVID-19 explained 11.3% of the total variance in well-being and 1.3% of the total variance in life satisfaction, and then well-being explained 19.4% of the total variance in life satisfaction'.

An interesting approach is that of Mehta [60], whose second research objective is meant to investigate the relationship between work from home (WFH) related to pandemic lockdown and employee happiness, building WFH on four constructs: autonomy, convenience, psychosocial safety and work participation, the latter predicting a 23.9% variance in perceived happiness. This study is particularly important because, along with other research papers, it supports the idea that once the world slowly returns to normal, getting closer to its pre-pandemic state, the WFH arrangements might actually be kept on the long run [10].

Stănculescu [78] validates the Romanian version of the Fear of COVID-19 Scale on a sample of 809 adults. Furthermore, this study finds a significant positive correlation between fear and stress or depression, and negative correlations with resilience and happiness. Happy people have an improved ability to handle stressful situations. In the United States, Wanberg et al. [82] investigate the level of psychological well-being in terms of life satisfaction and depressive symptoms during the COVID-19 pandemic. They use data from 2 surveys on 1433 individuals and show a nonlinear relationship with changes in well-being. Furthermore, people with the highest income levels experience a greater decrease in life satisfaction from before to during COVID-19 than people with lower income levels. Fu et al. [26] examine anxiety with the COVID-19 pandemic in the United States. They show that anxiety is associated with performance, engagement, and emotional exhaustion. In the same view, Gabriel et al. [27] investigates the anxiety of job seekers in the context of COVID-19 and how it is amplified for those who held higher levels of conspiracy theory beliefs.

Certain studies of the specialized literature focus on certain niche groups of people, to observe the effects of the pandemic upon them in particular. For example, Chen et al. [13] apply an online questionnaire tailored for adolescents, between February and November 2020, on issues related to their stress, certain psychosomatic symptoms, their happiness, their relations with parents and home life, social support and peers, their school environment, their duration of sleep and physical activity, and their general feeling and trust in future. Their results on almost 600 Swedish teenagers show that somehow, the individuals not exposed to COVID-19 present no differences in longitudinal changes in mental health, health behaviors and relationships with their social group than individuals exposed to COVID-19. Furthermore, Mansueto et al. [57] use an online survey of Italian healthcare workers to investigate their exposure to COVID-19, and its associated worries and life changes, while controlling for various sociodemographic variables and clinical ones as well. Then, Yamamura and Tsustsui [87] investigate the relationship between the closures of primary schools and that of junior high schools on the one hand and mothers’ mental health evolution on the other. The former worsens the mental health of mothers, while the latter improves it. Their study considers anger, fear, anxiety, and happiness as dependent variables and estimates them as a function of pandemic waves, primary/junior high school closure, and interactions between them, income, and age, on a short panel database from mid-March to mid-April 2020. In terms of future expectations, Hammarberg et al. [34] study the preferred policy options regarding post-COVID-19 mental health. The findings based on a 9220 people which answer at item “To have a publicly available plan about management of future pandemics” showed that 46.1% of respondents considers very helpful to have a plan.

It is obvious that not only health and well-being have declined throughout the world during the COVID-19 pandemic, but financial stability has also been severely affected. According to Wyplosz [86], national public debts would increase by approximately 15–30% of GDP and the entire post-pandemic European economy would be different. Indeed, increased public debts and their related fiscal and monetary implications have been addressed by Elyassi [21] and Zahariev et al. [92]. On the other hand, Laborde et al. [49] estimate that globally, more than 140 million people could fall into extreme poverty.

In the recent literature, the financial impact has been studied in terms of the market (e.g.: volatility in [2, 5, 22], business companies [3, 45], [48]) or at a microeconomic level [16, 59, 68]. There are findings showing that low-income individuals tend to be more impacted by pandemic (see [72], and also [38, 41], highest wealth were least likely to be financially impacted [41], or women are 24% more likely to permanently lose their jobs in the COVID-19 context [16]. Indeed, gender inequalities would worsen during any type of crisis according to Fisher & Ryan [24], being spread across various domains, such as health and well-being or work and poverty.

From a macroeconomic point of view, Elyassi [21] considers the COVID-19 pandemic to have arrived immediately after the world economy gained its strength back after the financial and economic crisis and the internal economic lessons that should have been learnt to rely on improved supervision for the real and nominal sectors of the market economy. This paper underlines the fact that many countries have made great public expenses throughout this difficult period, increasing their national public debts and further leading to austerity, decreased public expenses and raised taxes. According to this study, from the point of view of national monetary policies, countries would opt between decreased interest rates for keeping the cost of lending down, and increased interest rates, fearing price instabilities. Furthermore, Zahariev et al. [92] analyze the connection between fiscal and debt sustainability indicators for the European Union member states, covering the 2015–2019 time period and pandemic economic shocks’ implications as well. Fiscal reforms throughout Europe are outlined and the authors consider them to be urgent, due to this ‘unprecedented economic crisis’.

From the point of view of the challenges brought by the pandemic for business firms, Didier et al. [18] present the implications of companies’ so-called hibernation, meant to decrease their expenses to a bare minimal level and to appeal to credit resources for surviving the pandemic crisis. Their study carefully analyzes national policy measures on two groups: the ones related to loans, equities and guarantees on the one hand, and the ones related to public revenues and expenses on the other hand. Furthermore, Krűger and Meyer [48] deal with various national business environment stability policies and social policies as well, to prevent transmissions and help recoveries in a post-pandemic economic world. Basically, their study compares several European countries to South Africa from the point of view of their governmental policies meant to help businesses reduce their financial losses due to the spread of the pandemic (i.e., tax and bank payment holidays, grants, mortgage interruptions, VAT deferments, and others). Getting closer to small business owners, Marjanski & Sulkowski (2021) consider family businesses extremely sensitive to the threats posed by the pandemic related to the way of continuing their business operations, keeping their employees and a certain financial stability. Unlike larger companies, small family businesses do not hold previous know-how on responding to the effects of the crisis. Marjanski &Sulkowski [59] also study the relationship between the size of companies and their financial liquidities throughout the pandemic, noticing that small firms that have not been sufficiently liquid for the pandemic challenge have used national aids and reduced their fixed expenses, too. Nonetheless, Nguyen & Dinh [62] study Vietnamese businesses before (in 2019) and throughout the pandemic to conclude that the effective adoption of risk management tools has helped the companies’ financial ratios, providing them with an improved use of assets and increased liquidities, compared to companies that have just expressed their risk concerns. While considering debt management to be an efficient provider of economic stability in times of crisis, the authors support the need for ex ante risk management strategies for future crisis.

Getting closer to an individual type of approach, indeed studies showed that low income and unemployment decrease the mental health [58, 63, 76]. For instance, Nnawulezi & Hacskaylo [63] target their study on the employees of organizations that support survivors of intimate partner violence, whose main worries regarded a maintained health status for themselves, their colleagues and the survivors in their care, although the services they provide were not adapted to the imposed social distancing. Basically, the pandemic has brought several financial, social, and emotional repercussions to them as well. Then, Jones et al. [38] deal with a sample of New York students whose mental disorders and financial stresses are studied, on two levels of subjective assessments: at the beginning of last year’s first semester (prior to the pandemic) and during the pandemic. More than half of their respondents reported both anxiety (54.5%) and financial instability (54.1% for themselves and 68.9% for someone in their family) related to the COVID-19 pandemic. Some of the predictors of their anxiety and depression are their insecurities associated with the lack of food and housing and their close experiences with potential symptoms of COVID-19. Students identify a decreased ability to study as a result of the pandemic (56.8%) and general financial worries as well, such as the increase of their household-related expenditures (47.8% of respondents). Some students even report an increase in their alcohol, tobacco and marijuana consumption. Regarding substance abuse under pandemic conditions, an interesting paper is that of Siddiqi et al. [76], which studies smoking habits of individuals from Pakistan, a low-income country. Smoking habits have fluctuated since COVID-19 started as a function of their nicotine dependence and motivation to quit smoking and the financial variations in people's income.

Summing up, the COVID-19 pandemic has brought along important health and financial disorders for individuals and nations, in the context of a worldwide affected economy. Most people have found it difficult to cope with the pandemic crisis. Lopez et al. [55] focus on the role played by mindfulness before and during the lockdowns, supporting the idea of it bringing an ease on the negative implications of the pandemic. Psychological discomforts are indirectly related to people’s mindfulness profiles, controlling for sex, age, socioeconomic status (financial insecurities or ownership of a property) and housing privacy ratios. Nonetheless, the social media and the press have also had an important effect upon the way people reacted to the spread-out information. Park [67] uses semantic network analysis to study the frequency of certain pandemic-related key words on social media environments, throughout the first 6 months of the beginning of the pandemic. Anyway, although some of the specialized literature reports results on prior to COVID-19 and during COVID-19 periods, up to our knowledge, there is no study on the subjective perception of individuals upon their after COVID-19 life, well-being, and financial means, which gives an added value to our research.

## Data and methodology

### Variables

#### Dependent variable: Happiness

The Subjective Happiness Scale (SHS) is one of the most commonly used measures of subjective happiness. The SHS was developed by Lyubomirsky and Lepper [56] and is composed of four items: “two items ask respondents to characterize themselves using both absolute ratings and ratings relative to peers, while the other two items offer brief descriptions of happy and unhappy individuals and ask respondents to describe the extent to which each characterization describes them.” They use a 7-point Likert scale item that indicates the degree of subjective happiness, higher scores indicating greater happiness. The final composite score is computed as the average of the individual scores for each of the assessed dimensions, as follows:

1 $${\text{Happiness}} = \frac{{\sum\nolimits_{i = 1}^{4} {D_{i,j} } }}{4}$$

where *D*_*ij*_—score of dimension *D* for each *j* respondent (*i* is indexing the dimension to show summarization).

The psychometric properties of SHS have been examined in countries, such as the USA [56], Turkey [19], Malaysia [79], Mexico [71], China [61], Portugal [77], Italy [37], Greece [39], Romania [12] and others, with excellent or good internal consistency.

#### Independent variable: Financial stability

The independent variable financial stability is proxied by the following estimators: personal monthly income, income change, and family income change. In other words, financial stability incorporates the size of income, changes in personal and family income.

#### Control variables

As many factors affect the relationship between Happiness and Income, we must control for several factors to overcome the bias of omitted variables. There is an extensive literature on happiness but according to authors we have chosen the following control variables (Controls) reflecting the Socio-demographic status of individual: Gender [83, 91], Age [9], Education [88], and [14]), Urbanization, [11], Religion [14], Marital status ([35, 53], 14]. In addition, we check for the impact of Country of residence.

The description of the variables and their units and scales is presented in Table 1.

**Table 1:** Description of variables

Variables	Estimators	Description and way of calculation
*Dependent variable*		
Happiness	Subjective Happiness Scale	The Subjective Happiness Scale developed by Lyubomirsky and Lepper [56] is used. The scale ranges between 1 and 7 according to the responses to the following questions: For each of the following statements and/or questions, please circle the point on the scale that you feel is most appropriate to describe you • In general, I consider myself: 1 not a very happy person 7 a very happy person • Compared with most of my peers, I consider myself: 1 less happy 7 more happy • Some people are generally very happy. They enjoy life regardless of what is going on, getting the most out of everything. To what extent does this characterization describe you? 1 not at all 7 a great deal • Some people are generally not very happy. Although they are not depressed, they never seem as happy as they might be. To what extent does this characterization describe you? 1 not at all 7 a great deal
	Stress (for robustness checks)	The answers of respondents to the following question: Please evaluate your level of stress/anxiety (from 1 to 7 points): 1-low level 4-medium 7-high level
*Independent variable*		
Financial stability	Personal monthly income	The answers of respondents to the following question: Choose which is your personal monthly net income: Below 500 euros; Between 500 and 1000 euros; Between 1000 and 1500 euros; Between 1500 and 2000 euros; Between 2000 and 3000 euros; Over 3000 euros
	Income change	The answers of respondents to the following question: Has your monthly income changed in the context of COVID-19? Choose a variant: Yes, it has improved; Yes, it has gotten worse; No, it has stayed the same
	Family income change	The answers of respondents to the following question: In the context of COVID-19, has your family's income been affected? Choose a variant: Yes, it has improved; Yes, it has gotten worse; No, it has stayed the same
*Control variables*		
Socio-demographic status	Gender	Gender Male Female Other
	Age	The answers of respondents to the following question What is your age?
	Education	The answers of respondents to the following question: What is the highest level of education you have completed? Choose a variant Primary or secondary school; High school; Some college/University studies; Bachelors or equivalent; Masters/postgraduate studies; Doctoral level; Other
	Urbanization	The answers of respondents to the following question Which of the following best describes the area you live in? Choose a variant: Urban Rural
	Religion	The answers of respondents to the following question What is your religion? Choose a variant: Christian Catholic; Christian Orthodox; Christian Protestant; Other Christian; Judaism; Islam; Other religion; Atheist; Unaffiliated/ Nothing in particular; Prefer not to answer
	Marital status	The answers of respondents to the following question What is your marital status? Choose a variant: Single; In a relationship/engaged; Married; Divorced; Widow/ Widower; Other
	Country of residence	The answers of respondents to the following question Which is your country of residence?
*Repeated measurements—time assessment*		
Time	Time–Covid	Is the time variable, automatically constructed in the analysis procedure, that accounts for the time passing within the three measurement moments: pre, during and post COVID-19 pandemic

Source: own processing

### Methodology

**Fig. 1:**
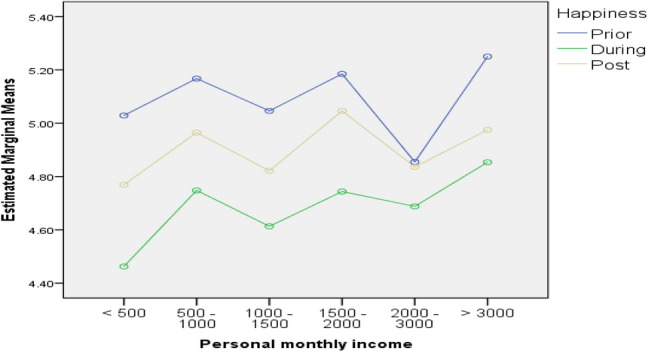
Profile plot: Marginal means—Happy vs. Personal income Source: own construction in SPSS 24

**Fig. 2:**
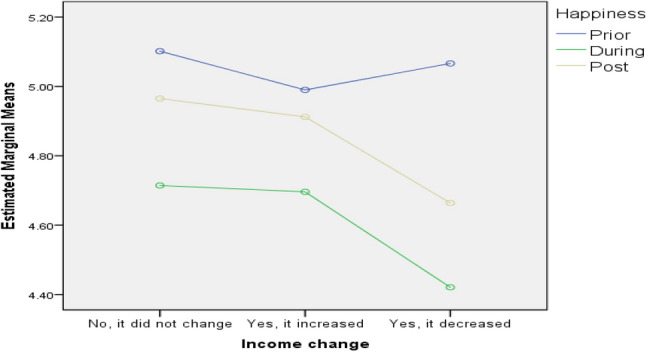
Profile plot: Marginal means—Happy vs. Income change Source: own construction in SPSS 24

**Fig. 3:**
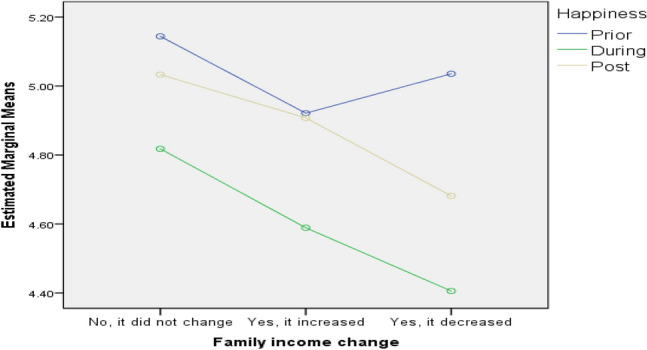
Profile plot: Marginal means—Happy vs. Family income change Source: own construction in SPSS 24

Following the standard data analysis procedures, variables were, first, descriptively assessed. We computed frequencies and percent for nominal and ordinal data and constructed the bar chart to visualize the features of such variables. Descriptive statistics along with normality tests and plots were applied on scale variables. However, since we have a large sample (1572 respondents), we can act under the Central Limit Theorem and both parametric and non-parametric procedures return similar results. Changes in happiness pre-, during, and post COVID-19 were evaluated using the Paired Samples *T*-test. The impact of the factor considered was first evaluated based on the simple ANOVA approach. In the last part of the analysis, we employed the GLM estimation in the Repeated Measures form to assess the impact of both time and the factors considered on the perceived happiness level. Profile plots were constructed to assess the marginal means of the dependents of the factor groups (financial stability) (Figs. 1, 2, 3 in Appendix A). We present both effects between subjects and effects and contrasts within subjects to evaluate the impact of COVID-19 and the financial factors on the level of perceived happiness. The between-subjects effects show how much the considered factors determine differences in the respondents in respect to the dependent variable. The within-subjects effects deal with the variability in time for a specific individual. As in the case of all factors, the Mauchly Sphericity test rejects the circularity of the variance–covariance matrix for the dependent variables, we ignore the Sphericity Assumed procedure and present the results of the Greenhouse–Geisser test (GG).

The last step is to evaluate the stability and robustness of the results. For this, on the one hand, we introduce control variables in the GLM estimations. On the other hand, we replace happiness with the level of perceived stress prior, during, and after COVID-19. In both cases, we follow the same analysis steps.

Analyses were conducted in SPSS 24 and Tableau Desktop 2021.3.6.

### Sample description

**Fig. 4:**
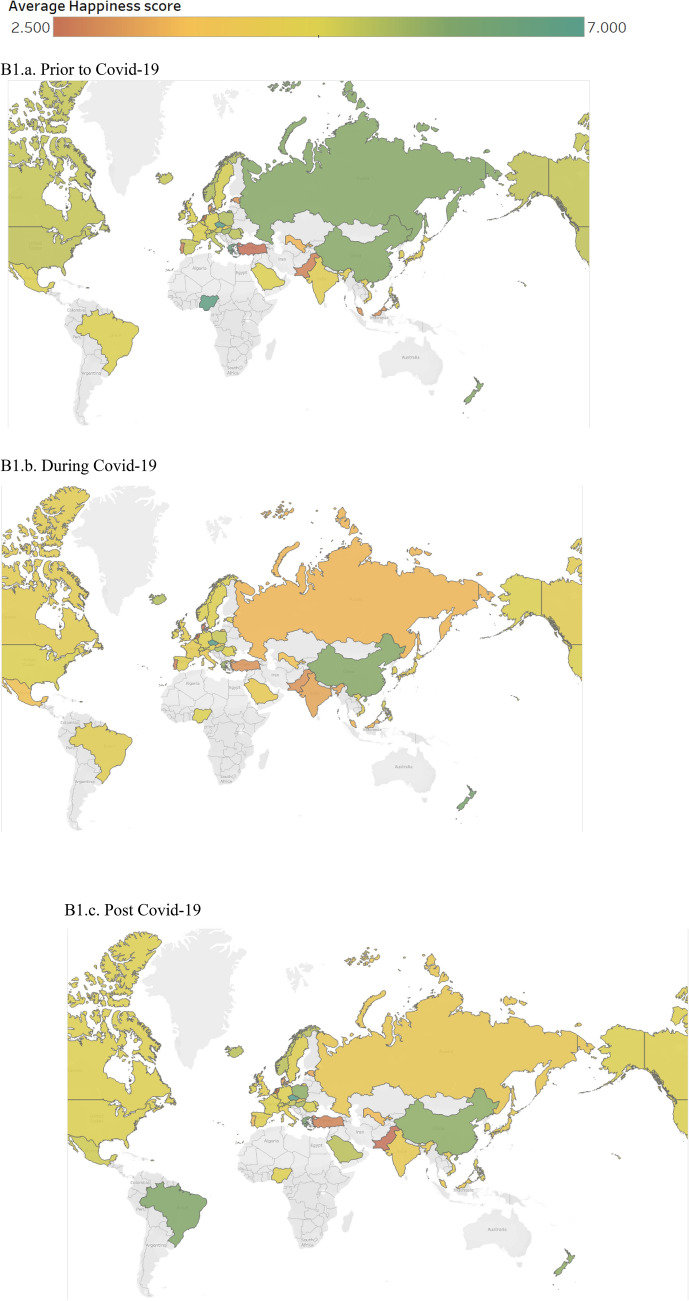
Map of average Happiness score. **a** Prior to COVID-19. Source: own construction in Tableau 2021.3.6 **b**During COVID-19. Source: own construction in Tableau 2021.3.6 **c**Post COVID-19. Source: own construction in Tableau 2021.3.6

After all quality adjustment procedures, a final sample of 1572 respondents is kept for the analysis, covering individuals living in 43 worldwide countries (see maps in Figure 4 in Appendix B). The questionnaires were addressed during the period May 2020 and July 2021. The sample is made up of 20% men and 80% women, most of whom live in urban areas (almost 78%). About half of the respondents are married, 27% in a relationship, and 23% are single, while the rest are divorced or widow. Regarding education, we observe a high educational level for our sample, since 30.4% of the respondents have a master’s degree, 27.7% a university degree and almost 10% a Ph.D. degree. Their average age is 32.8, with a standard deviation of 11.56 and a median of 30.

### Transparency and openness

We describe our sampling plan, all data exclusions (if any), all manipulations, and all measures in the study. All data are available as supplementary material, whereas analysis codes, and research materials are available upon request. Data were analyzed using IBM SPSS, version 24. This study’s design and its analysis were not preregistered.

## Results and discussion

### Main results

The simple descriptive assessment of the perceived happiness for the three periods considered, prior, during, and after the COVID-19 pandemic, shows that people felt happier before the virus appeared (average score of 5.0856—see Table 2). As expected, the lowest level of happiness is obtained for the actual time of the pandemic (4.6245), with a slight recovery afterward. This change can also be observed when comparing the average happiness score/ country in the three maps in Fig. 4 (a: a prior to COVID-19, b during the pandemic and c after it). In most cases, the average happiness level decreases during the pandemic (Fig. 4b), and it recovers afterward (Fig. 4c). But, in most cases, the perceived post COVID-19 happiness level is appreciated to be lower than the initial one. There are some exceptions. First, we see a similar happiness level in China and the Czech Republic all throughout the analyzed time span. Additionally, there are some more optimistic countries that have a higher average score for the post-pandemic situation. Respondents from Saudi Arabia, Mexico or Brazil consider they will be happier when the pandemic will be over.

**Table 2:** Descriptive statistics for happiness on the three periods assessed

	Mean	*N*	Std. deviation	Std. error mean
Happiness prior to COVID-19	5.0856	1572	1.22276	0.03084
Happiness in the context of COVID-19	4.6245	1572	1.30518	0.03292
Happiness post COVID-19	4.8713	1572	1.25584	0.03167

Source: own calculations in SPSS 24

Helliwell et al. [36] in *World Happiness Report* present difference between 2020 of subjective well-being and their main determinants and 2017–2019 period. Unfortunately, Romania is not included in the database, but it can be seen both, in the world and in European countries, how the pandemic has worsened people's lives even if there are some exceptions.

The paired analysis shows that the differences are significant, with the highest gap level of 4.461 for happiness before COVID-19 and in the context of it (Table 3). Similary, Greyling et al. [32] examined happiness lost level in lockdown versus no lockdown period and compare the likelihoods (17–26% to be happy).

**Table 3:** Comparison analysis—happiness level

Paired samples test					
	Paired differences			*t*	Sig
	Mean	Std. dev	Std. error mean		
Pair 4					
Happiness prior to COVID-19—Happiness in the context of COVID-19	0.461	0.95217	0.02402	19.198	0.000
Pair 5					
Happiness prior to COVID-19—Happiness post COVID-19	0.214	0.78432	0.01978	10.829	0.000
Pair 6					
Happiness in the context of COVID-19—Happiness post COVID-19	−0.247	0.67137	0.01693	−14.576	0.000

Source: own calculations in SPSS 24

In terms of happiness, the analysis has pointed out a much higher level before the appearance of the COVID-19 pandemic compared to the current context and expectations about the post-COVID-19 pandemic. This result points out the significant impact the present pandemic has had on the individuals. Our results is in line with Dwidienawati et al. [20] which showed how happiness has deteriorated during mobility restriction (58%) in pandemic period.

Just as presented in the literature review part, there is a significant number of factors that led to this evolution of the individual happiness. As this study focuses on the impact of financial stability, altered or not by the present pandemic, we introduce the three proxies into analysis. The visual assessment of perceived happiness presented in Figs. 1, 2, 3 (see Appendix A) clearly shows that, regardless of the financial stability proxy, the highest means are attributed to the prior COVID-19 period. The related descriptive statistics are presented in Tables 14, 15 and 16 in Appendix C. People feel they were happier before the COVID-19 pandemic. However, we can see that there is an optimistic perception that things could get back toward normality and toward almost the same happiness level as before (the brown line in Figs. 1, 2, 3, from Appendix A). As expected, all the respondents have been very much affected by the COVID-19 pandemic, as we can see very low scores for this period. Regarding personal income, the profile plot shows that, while before the pandemic most income groups had relatively similar happiness levels, except for the 2000–3000 EUR/ month group, during the pandemic, people with lower incomes are more affected. This group of respondents is the most pessimistic, and the impact of the COVID-19 crisis will be felt on a longer time frame, as their average happiness score is the lowest even after the end of the pandemic. Additionally, this is the group for which we have the highest discrepancy between the perceived level of happiness before versus during and after the COVID-19 pandemic.

With respect to the income change, we observe that, definitely, a decrease in income led to lower happiness. The same specificities are to be found when the family income change is assessed (Fig. 3, Appendix A). Additionally, we can observe a higher difference in the perceived level of happiness during the pandemic between respondents whose families’ incomes are not affected and the ones that registered increases. It is peculiar to see that people with an increase in the family income during the crisis are feeling much unhappier than those with no registered change.

To assess the validity of the aspects depicted from the profile plots, we continue our analysis by assessing an individual impact of the financial stability factors upon happiness in each of the three periods of time. Table 4 shows that income, in any of the considered forms (personal or family), does not influence the level of happiness before the appearance of Covid-19.

**Table 4:** Happiness vs financial factors—ANOVA analysis synthesis

Variable	Happiness prior to COVID-19		Happiness in the context of COVID-19		Happiness post COVID-19	
	*F*	Sig	*F*	Sig	*F*	Sig
Personal monthly income	1.81	0.108	3.34	0.005	1.95	0.084
Income change	0.37	0.689	8.41	0.000	9.48	0.000
Family income change	2.16	0.115	19.06	0.000	14.9	0.000

Source: own calculations in SPSS 24

According to the literature, results are mixed: income seems to buy happiness [1, 25] or income buys little happiness [69]. It appears that the recent pandemic has led to a higher level of awareness of the need for financial stability of individuals, and the pandemic has affected the reported level of happiness.

The next step of our analysis is to evaluate the actual impact of the factors not only on each type of happiness measured, but on the overall variation in time.

Results in Table 5 show that the COVID-19 pandemic has significantly influenced the level of happiness (all intercepts are highly significant and have very large Eta^2^ values). Regarding the financial variables considered, variations in the level of happiness prior, during, and after the COVID-19 pandemic are significantly influenced by changes in personal and family income (when measured if stable, increasing, or decreasing), and the actual personal income. Among the significant factors, family income change has the highest impact, with the highest Eta^2^ value (0.015).

**Table 5:** Variance analysis—happiness between-subjects effects

Model	Source	*F*	Sig	Partial Eta^2^
1	Intercept	15136.9	0.000	0.906
	Personal monthly income	2.35	0.039	0.007
2	Intercept	9292.9	0.000	0.856
	Income change	5.3	0.005	0.007
3	Intercept	9493.4	0.000	0.858
	Family income change	11.73	0.000	0.015

Source: own calculations in SPSS 24

All of the within-subjects effects are highly significant, a fact that proves their contribution to the model. When the polynomial contrasts are constructed, we can see that the within-subjects effects of Time–COVID (prior, during, and post) are highly significant both in the linear and quadratic form. Additionally, the joint effects of the Time–COVID * factor are as follows:

- highly significant in both linear and quadratic forms for personal and family income change;
- significant at 1% in the quadratic form and at 10% in the linear form for personal monthly income.

Consequently, we may conclude that the used financial proxies lead to two types of variations in the analyzed sample: (1) between the groups given by the factors (significant between effects), and (2) in time, from prior to post-COVID-19 (within effects).

In the last part of this research, we evaluate the stability of the results, in two ways, as explained in the methodological part.

First, we introduce the *control variables* in the analysis. The time variation present in the model is significantly contributing to the model, both in the between- and within-effects forms (in almost all cases, except for gender, which changes the probability to values > 0.1—see Tables 6, 7 and 8). With respect to the financial stability variables considered in this analysis as factors of influence for the individual well-being proxied by the level of happiness, we can see that their effect is no longer manifesting in both between- and within-forms in all models once control variables are introduced (see Tables 6, 7 and 8). Consequently, the perceived impact of the changes in financial stability upon the happiness level is altered/conditioned by the sociodemographic control variables.

**Table 6:** Variance analysis—hapiness within-subjects analysis

Model	Source	Effects—GG test		Contrasts			
				Linear		Quadratic	
		*F*	Sig	*F*	Sig	*F*	Sig
1	Time–COVID	108.0	0.000	49.0	0.000	160.1	0.000
	Time–COVID *Personal monthly income	2.7	0.004	1.9	0.095	3.5	0.004
2	Time–COVID	81.9	0.000	38.2	0.000	119.8	0.000
	Time–COVID *Income change	12.4	0.000	20.3	0.000	5.6	0.004
3	Time–COVID	80.2	0.000	23.3	0.000	129.7	0.000
	Time–COVID *Family income change	16.2	0.000	20.9	0.000	12.03	0.000

Source: own calculations in SPSS 24

**Table 7:** Variance analysis—happiness between-subjects effects with control factors

Model	Source	Control factor (Sig.)						
		Age	Gender	Education	Urban	Religion	Marital status	Country
1	Time–COVID (intercept)	0.000	0.000	0.000	0.000	0.000	0.000	0.000
	Personal monthly income	0.027	0.229	0.363	0.026	0.715	0.455	0.122
	Control factor	0.330	0.965	0.324	0.246	0.000	0.10	0.308
2	Time–COVID (intercept)	0.000	0.000	0.000	0.000	0.000	0.000	0.000
	Income change	0.005	0.233	0.738	0.045	0.453	0.327	0.114
	Control factor	0.912	0.834	0.086	0.324	0.364	0.038	0.419
3	Time–COVID (intercept)	0.000	0.000	0.000	0.000	0.000	0.000	0.000
	Family income change	0.000	0.139	0.600	0.004	0.134	0.918	0.012
	Control factor	0.556	0.911	0.095	0.249	0.374	0.174	0.355

Source: own calculations in SPSS 24

**Table 8:** Happiness—within-subjects analysis

Model	Source	Control factor (GG test Sig.)						
		Age	Gender	Education	Urban	Religion	Marital status	Country
1	Time–COVID (intercept)	0.000	0.201	0.000	0.000	0.000	0.000	0.000
	Personal monthly income	0.048	0.002	0.042	0.178	0.032	0.233	0.460
	Control factor	0.003	0.001	0.018	0.448	0.011	0.000	0.904
2	Time–COVID (intercept)	0.000	0.236	0.000	0.000	0.000	0.000	0.031
	Income change	0.000	0.013	0.016	0.000	0.027	0.016	0.088
	Control factor	0.000	0.006	0.197	0.013	0.001	0.054	0.509
3	Time–COVID (intercept)	0.000	0.188	0.000	0.000	0.009	0.000	0.006
	Family income change	0.000	0.011	0.074	0.000	0.001	0.118	0.734
	Control factor	0.001	0.009	0.372	0.632	0.000	0.052	0.779

Source: own calculations in SPSS 24

Age is not impacting Happiness when the between-effects are assessed (Table 7), but it is highly significant in the within-effects (Table 8). Additionally, financial stability is highly significant in all Age models, with both types of effects. It is interesting to see that most of the control factors are not significant in the between-effects assessment (Table 7), but many of them become significant in the within-effects analysis (Table 8). The same results as for Age are obtained for Urban in the between-analysis, but some changes appear in the within-form. When controlling for where the person lives, the time change in happiness level is not due anymore to the personal monthly income. Gender, Education, Marital status, and Country have similar results and they all alter the significance of the financial stability proxies. There is only one exception, for Family income change controlled by Country. Things remain quite similar for Country in the within-effects assessment, but with an insignificant Family income change. For the other three variables, both the significant effects of the financial stability proxies and theirs preserve. Religion has an interesting effect, in the sense that there are significant differences in the perceived happiness level of different religious groups together with personal income, but not together with changes in personal or family income. But financial stability highly impacts the happiness level changes when controlling for religion in the within-effects evaluation.

We can conclude that the sociodemographic variables have a significant impact in the time change of the happiness level perception, rather than a purely between groups one.

Taking into account the fact that we use three proxies for financial stability and there are always more of them significantly contributing to the model, regardless of the control factor used, we may conclude that our results are stable and valid. As such, financial stability is significantly influencing the level of perceived well-being and its variation in time given by the COVID-19 pandemic. Consequently, financial instability due to the COVID-19 pandemic adds to the negative psychological impact that all restrictive measures caused by this situation had upon the individual. The impact is perceived in the long term, as most of the respondents are pessimistic and consider their financial status will not be the same after the pandemic, but worser than before it. Out of these financial factors, income change is the most significant, regardless of the control factor used. Our results are in line with previous research conducted by Gall et al. [28] who showed that mean reduction in well-being was associated with financial instability and mental health comorbidity. Also, VanderWeele et al. [80] showed in the USA how well-being has declined before and during the COVID-19 in terms of financial stability, happiness and health.

### Robustness checks

For robustness checks, we replace happiness by stress and apply the same type of methodology. From Table 9, we may see that the level of perceived stress before the COVID-19 pandemic is statistically insignificant. Regardless of the period assessed, any change in the income status of the respondents leads to stress. Consequently, we may conclude that financial status and changes in the individual’s life significantly influence the perceived levels of happiness and stress during and after the COVID-19 pandemic. In a longitudinal case study in the Netherlands, Kok et al. [47], showed that the symptoms of depressive, anxiety, and worry were stable since April–May, but loneliness feelings increased.

**Table 9:** Stress vs economic and financial factors—comparison analysis synthesis

Variable	Stress prior to COVID-19		Stress in the context of COVID-19		Stress post COVID-19	
	*F*	Sig	*F*	Sig	*F*	Sig
Personal monthly income	0.943	0.452	4.33	0.001	4.29	0.001
Income change	0.307	0.736	6.03	0.002	7.67	0.000
Family income change	0.393	0.675	18.67	0.000	15.86	0.000

Source: own calculations in SPSS 24

The stability of the main results is also confirmed by the repeated measurements estimations. The variance analysis presented in Table 10 shows the same type of influences—all factor variables significantly contributed to changes in the level of Stress, just like in the case of Happiness, with Family income change having, once again, the highest impact measured by Eta^2^ (0.015—among all three main factors).

**Table 10:** Variance analysis—stress between-subjects effects

Model	Source	*F*	Sig	Partial Eta^2^
1	Intercept	6219.8	0.000	0.799
	Personal monthly income	3.33	0.005	0.011
2	Intercept	4109.2	0.000	0.724
	Income change	5.02	0.007	0.006
3	Intercept	4128.8	0.000	0.725
	Family income change	11.63	0.000	0.015

Source: own calculations in SPSS 24

Consequently, we may conclude that the considered financial factors significantly impact the perceived well-being of individuals measured both through happiness and stress, before, during and after the COVID-19 pandemic.

But just like in the case of Happiness, we also include control variables when Stress is the proxy for subjective well-being. We obtain similar results with respect to time: time variation in the perceived stress level is highly significant. Thus, we may conclude that the variation of stress is significantly influenced by the reference period—prior, during, and post COVID-19 pandemic (Table 11).

**Table 11:** Stress—within-subjects analysis

Model	Source	Effects–GG test		Contrasts			
				Linear		Quadratic	
		*F*	Sig	*F*	Sig	*F*	Sig
1	Time–COVID	209.9	0.000	136.8	0.000	290.5	0.000
	Time–COVID *Personal monthly income	3.3	0.001	4.53	0.000	1.94	0.085
2	Time–COVID	161.2	0.000	117.9	0.000	209.05	0.000
	Time–COVID *Income change	4.6	0.002	7.08	0.001	1.87	0.155
3	Time–COVID	152.3	0.000	95.42	0.000	215.03	0.000
	Time–COVID *Family income change	13.99	0.000	17.6	0.000	10.03	0.000

Source: own calculations in SPSS 24

When control variables are, once again, introduced in the assessment, we may see that results are quite similar with the happiness situation in the case of Gender, Urban, Religion, Marital status or Country (Tables 12 and 13).

**Table 12:** Variance analysis—stress between-subjects effects with control factors

Model	Source	Control factor (Sig.)						
		Age	Gender	Education	Urban	Religion	Marital status	Country
1	Time-Covid (intercept)	0.000	0.000	0.000	0.000	0.000	0.000	0.000
	Personal monthly income	0.079	0.367	0.017	0.051	0.405	0.600	0.066
	Control factor	0.001	0.059	0.005	0.138	0.552	0.789	0.779
2	Time-Covid (intercept)	0.000	0.000	0.000	0.000	0.000	0.000	0.000
	Income change	0.012	0.557	0.898	0.064	0.289	0.726	0.078
	Control factor	0.000	0.282	0.004	0.266	0.828	0.952	0.595
3	Time-Covid (intercept)	0.000	0.000	0.000	0.000	0.000	0.000	0.000
	Family income change	0.000	0.072	0.502	0.001	0.181	0.356	0.235
	Control factor	0.000	0.147	0.023	0.186	0.870	0.878	0.767

Source: own calculations in SPSS 24

**Table 13:** Stress within-subjects analysis

Model	Source	Control factor (GG test Sig.)						
		Age	Gender	Education	Urban	Religion	Marital status	Country
1	Time–COVID (intercept)	0.000	0.092	0.000	0.000	0.000	0.000	0.000
	Personal monthly income	0.000	0.018	0.497	0.063	0.781	0.709	0.307
	Control factor	0.234	0.000	0.850	0.383	0.452	0.005	0.498
2	Time–COVID (intercept)	0.000	0.143	0.000	0.000	0.000	0.000	0.000
	Income change	0.002	0.112	0.128	0.096	0.205	0.019	0.709
	Control factor	0.335	0.000	0.289	0.155	0.056	0.067	0.087
3	Time–COVID (intercept)	0.000	0.134	0.000	0.000	0.001	0.000	0.000
	Family income change	0.000	0.081	0.000	0.000	0.148	0.003	0.397
	Control factor	0.490	0.000	0.421	0.919	0.000	0.648	0.016

Source: own calculations in SPSS 24

The impact of Age or Education changes dramatically and becomes highly significant in the between-effects case, and insignificant in the within- form. This implies that there are significant differences in the perceived stress level for different ages and education levels, but these differences do not impact the time variation of these perceptions.

Comparative evaluation of the stress and happiness levels conditioned by the COVID-19 shows that the first is more intensely affected. The perceived level of stress increases by more than 30%, while the decrease in the perceived level of happiness is below 10% (prior versus during the pandemic).

## Conclusions

This is one of the first studies to investigate the relationship between happiness and income before, during, and after the COVID-19 pandemic. In terms of future expectations, basically to get at one point in future to talk about COVID-19 pandemic as a past event or to talk about it as a common virus, our study contributes to understanding how income sensitivity may cause changes in happiness reporting. As expected, well-being during COVID-19, as compared to the previous period, has decreased, while in future, people expect to be happier, but not more than in the past when they did not know about the existence of this virus. This is a very important result, showing the intensity of the impact this pandemic had upon the individual. Financial pressure added to the negative impact all the restrictions had in such a manner as people are more pessimistic about their future, both in terms of only happiness, and in terms of their financial stability. In this research, we apply a global assessment on respondents from different countries. This means that, on average, regardless of their nationality, ethnicity, place of living, etc., people are worried about their financial stability, and it significantly impacts their perceived happiness level. We show that religion, which is correlated to spatial positioning, is providing significant effects in the models, conditioning the relationship between happiness and financial stability.

Even if recent literature suggests that money does not buy happiness, we continue to emphasize the importance of money in achieving happiness. Our results show that both the size of income and changes in personal or family income affect the levels of happiness or stress reported by the individuals, worldwide.

These findings are important for policymakers to improve the conditions of living in the areas of health and financial stability. Our results also suggest that a change in personal income may disrupt the happiness level of a family member; respectively, it may be a factor that directly acts upon one’s stress level. Stress and happiness are especially important for mental health. If people are happy and peaceful, they are more likely to report different coping skills. Future research should examine how governments can help increase the coping ability in the context of a pandemic in which financial instability exists for a large part of the population. This is very important as the present situation in some parts of the world is showing us desperate decisions of citizens due to these aspects.

Additionally, we point out that there are additional sociodemographic aspects that impact both the happiness and the stress level, such as age, education, religion, marital status, etc.

Our research has some general limitations despite its strengths. First, in our study, some of the variables are assessed with self-reports, which increase the subjectiveness level. Second, our results may have partial generalizability across cultures around the world. This, because we are applying a global assessment. Future research implies, from this perspective, to include spatial effects and assess spatial differences that may appear in attitudes and perceptions around the world. Such a spatial evaluation would contribute even more by providing governments, authorities and organizations working in the field new information on how to cope with these effects conditioned by local specificities.

Third, our questionnaire has not included and validated any positive coping scale. However, this is an opportunity and, at the same time, a duty for future work to further investigate the impact of the pandemic on public health. It remains that in future, we will use longitudinal studies to measure post-COVID happiness in real terms rather than expectations, when the COVID-19 pandemic remains a black spot in human history.

Our methodological approach is a standard one, based on classical estimation methods. As we extend the sample size and include spatial effects, we will also turn toward more modern data analysis procedures. We intend to use Machine Learning/ Artificial Intelligence techniques to validate the present results. Out of these, there are, on the one hand, estimation methods, and on the other, sentiment analysis tools that provide us means to build sentiment indexes.

### Electronic supplementary material

Below is the link to the electronic supplementary material.

Supplementary file1 (SAV 158 KB)

Supplementary file2 (XLSX 266 KB)

Supplementary file3 (XLSX 290 KB)

## Data Availability

All the data provided from public database.

## Appendices

### Appendix A: Profile plots to assess the marginal means of the dependents of the factor groups (financial stability)

See Figs. 1, 2 and 3.

### Appendix B: Map of happiness

See Fig. 4.

### Appendix C: Descriptive statistics

See Tables.

**Table 14:** Descriptive statistics for happiness and personal monthly income

	*N*	Mean	Std. dev
Happiness prior to COVID-19			
< 500	578	5.0290	1.27379
500–1000	485	5.1675	1.17344
1000–1500	227	5.0463	1.16861
1500–2000	119	5.1849	1.22883
2000–3000	93	4.8548	1.26422
> 3000	70	5.2500	1.19707
Total	1572	5.0856	1.22276
Model fixed effects			1.22119
Happiness in the context of COVID-19			
< 500	578	4.4628	1.33872
500–1000	485	4.7479	1.28836
1000–1500	227	4.6134	1.21275
1500–2000	119	4.7437	1.33541
2000–3000	93	4.6882	1.35015
> 3000	70	4.8536	1.20348
Total	1572	4.6245	1.30518
Model fixed effects			1.30034
Happiness post COVID-19			
< 500	578	4.7695	1.31752
500–1000	485	4.9649	1.21684
1000–1500	227	4.8216	1.13672
1500–2000	119	5.0462	1.22862
2000–3000	93	4.8360	1.27821
> 3000	70	4.9750	1.33863
Total	1572	4.8713	1.25584
Model fixed effects			1.25395

Source: own calculations in SPSS 24

^14^,

**Table 15:** Descriptive statistics for happiness and income change

	*N*	Mean	Std. dev
Happiness prior to COVID-19			
No, it did not change	1022	5.1015	1.21184
Yes, it increased	74	4.9899	1.23133
Yes, it decreased	476	5.0662	1.24619
Total	1572	5.0856	1.22276
Model fixed effects			1.22325
Happiness in the context of COVID-19			
No, it did not change	1022	4.7143	1.26954
Yes, it increased	74	4.6959	1.18298
Yes, it decreased	476	4.4207	1.37655
Total	1572	4.6245	1.30518
Model fixed effects			1.29907
Happiness post COVID-19			
No, it did not change	1022	4.9650	1.21991
Yes, it increased	74	4.9122	1.20105
Yes, it decreased	476	4.6639	1.31657
Total	1572	4.8713	1.25584
Model fixed effects			1.24911

Source: own calculations in SPSS 24

^15^ and

**Table 16:** Descriptive statistics for happiness and family income change

	*N*	Mean	Std. dev
Happiness prior to COVID-19			
Yes, it increased	73	4.9212	1.22820
Yes, it decreased	697	5.0355	1.23340
Total	1572	5.0856	1.22276
Model fixed effects			1.22186
Happiness in the context of COVID-19			
No, it did not change	802	4.8180	1.26883
Yes, it increased	73	4.5890	1.17510
Yes, it decreased	697	4.4057	1.32599
Total	1572	4.6245	1.30518
Model fixed effects			1.29043
Happiness post COVID-19			
No, it did not change	802	5.0330	1.22302
Yes, it increased	73	4.9075	1.20654
Yes, it decreased	697	4.6815	1.27337
Total	1572	4.8713	1.25584
Model fixed effects			1.24487

Source: own calculations in SPSS 24

^16^
